# COVID-19 Booster Vaccination in Early Pregnancy and Surveillance for Spontaneous Abortion

**DOI:** 10.1001/jamanetworkopen.2023.14350

**Published:** 2023-05-19

**Authors:** Elyse O. Kharbanda, Jacob Haapala, Heather S. Lipkind, Malini B. DeSilva, Jingyi Zhu, Kimberly K. Vesco, Matthew F. Daley, James G. Donahue, Darios Getahun, Simon J. Hambidge, Stephanie A. Irving, Nicola P. Klein, Jennifer C. Nelson, Eric S. Weintraub, Joshua T. B. Williams, Gabriela Vazquez-Benitez

**Affiliations:** 1 HealthPartners Institute, Minneapolis, Minnesota; 2 Department of Obstetrics and Gynecology, Weill Cornell Medical College, New York, New York; 3 Kaiser Permanente Center for Health Research, Portland, Oregon; 4 Institute for Health Research, Kaiser Permanente Colorado, Denver; 5 Marshfield Clinic Research Institute, Marshfield, Wisconsin; 6 Kaiser Permanente Southern California, Pasadena; 7 Denver Health, Denver, Colorado; 8 Kaiser Permanente Vaccine Study Center, Oakland, California; 9 Kaiser Permanente Washington Health Research Institute, Seattle, Washington; 10 Centers for Disease Control and Prevention, Atlanta, Georgia

## Abstract

**Importance:**

Adherence to COVID-19 booster vaccine recommendations has lagged in pregnant and nonpregnant adult populations. One barrier to booster vaccination is uncertainty regarding the safety of booster doses among pregnant people.

**Objective:**

To evaluate whether there is an association between COVID-19 booster vaccination during pregnancy and spontaneous abortion.

**Design, Setting, and Participants:**

This observational, case-control, surveillance study evaluated people aged 16 to 49 years with pregnancies at 6 to 19 weeks’ gestation at 8 health systems in the Vaccine Safety Datalink from November 1, 2021, to June 12, 2022. Spontaneous abortion cases and ongoing pregnancy controls were evaluated during consecutive surveillance periods, defined by calendar time.

**Exposure:**

Primary exposure was receipt of a third messenger RNA (mRNA) COVID-19 vaccine dose within 28 days before spontaneous abortion or index date (midpoint of surveillance period in ongoing pregnancy controls). Secondary exposures were third mRNA vaccine doses in a 42-day window or any COVID-19 booster in 28- and 42-day windows.

**Main Outcomes and Measures:**

Spontaneous abortion cases and ongoing pregnancy controls were identified from electronic health data using a validated algorithm. Cases were assigned to a single surveillance period based on pregnancy outcome date. Eligible ongoing pregnancy time was assigned to 1 or more surveillance periods as an ongoing pregnancy-period control. Generalized estimating equations were used to estimate adjusted odds ratios (AOR) with gestational age, maternal age, antenatal visits, race and ethnicity, site, and surveillance period as covariates and robust variance estimates to account for inclusion of multiple pregnancy periods per unique pregnancy.

**Results:**

Among 112 718 unique pregnancies included in the study, the mean (SD) maternal age was 30.6 (5.5) years. Pregnant individuals were Asian, non-Hispanic (15.1%); Black, non-Hispanic (7.5%); Hispanic (35.6%); White, non-Hispanic (31.2%); and of other or unknown (10.6%); and 100% were female. Across eight 28-day surveillance periods, among 270 853 ongoing pregnancy-period controls, 11 095 (4.1%) had received a third mRNA COVID-19 vaccine in a 28-day window; among 14 226 cases, 553 (3.9%) had received a third mRNA COVID-19 vaccine within 28 days of the spontaneous abortion. Receipt of a third mRNA COVID-19 vaccine was not associated with spontaneous abortion in a 28-day window (AOR, 0.94; 95% CI, 0.86-1.03). Results were consistent when using a 42-day window (AOR, 0.97; 95% CI, 0.90-1.05) and for any COVID-19 booster in a 28-day (AOR, 0.94; 95% CI, 0.86-1.02) or 42-day (AOR, 0.96; 95% CI, 0.89-1.04) exposure window.

**Conclusions and Relevance:**

In this case-control surveillance study, COVID-19 booster vaccination in pregnancy was not associated with spontaneous abortion. These findings support the safety of recommendations for COVID-19 booster vaccination, including in pregnant populations.

## Key Points

**Question:**

Is COVID-19 booster vaccination in early pregnancy associated with an increased risk of spontaneous abortion?

**Findings:**

In this case-control surveillance study of more than 100 000 pregnancies at 6 to 19 weeks’ gestation from 8 health systems in the Vaccine Safety Datalink, the odds of having received a COVID-19 booster vaccination in either a 28- or 42-day exposure window before spontaneous abortion were not increased compared with ongoing pregnancies.

**Meaning:**

These findings support the safety of COVID-19 booster vaccination in early pregnancy.

## Teaser

This cross-sectional study assesses whether COVID-19 booster vaccination of pregnant people is associated with spontaneous abortion.

## Introduction

As of March 2023, more than 682 million SARS-CoV-2 infections and more than 6.8 million COVID-19 deaths have been reported worldwide.^1^ Although COVID-19 in otherwise healthy young adults is often mild or asymptomatic, infections during pregnancy are associated with increased risk of morbidity and adverse birth outcomes.^2,3^ Vaccination has been shown to reduce the risks of severe disease during pregnancy and to provide additional protection from complications in newborns.^4,5,6,7,8,9^

Because of waning immunity and the emergence of more contagious variants, starting in September 2021, booster doses of the messenger RNA (mRNA) vaccines were made available for populations in the United States who had completed the primary vaccine series and were at increased risk for severe illness due to COVID-19, including pregnant people.^10^ Subsequently, all adolescents and adults were encouraged to receive a COVID-19 vaccine booster after primary vaccination.^11^

Adherence to COVID-19 booster vaccine recommendations has lagged in pregnant and nonpregnant adult populations.^12,13^ One barrier to booster vaccination is the uncertainty regarding the effectiveness and duration of protection of additional vaccine doses. Others may question whether a booster is needed given a prior history of SARS-CoV-2 infection. An additional concern among pregnant people is regarding the safety of booster doses. Previous studies^5,14,15,16,17^ have demonstrated that receipt of 1 or 2 mRNA COVID-19 vaccine doses in pregnancy, as part of the primary vaccine series, was not associated with adverse pregnancy or birth outcomes, including spontaneous abortion, preterm birth, small-for-gestational-age birth, and infant neonatal care admissions. The aim of the current study was to evaluate potential associations between COVID-19 booster vaccination in early pregnancy and spontaneous abortion through adaptation of previously described COVID-19 vaccination in pregnancy safety surveillance.^15^

## Methods

The Vaccine Safety Datalink (VSD) is a collaborative effort between the Centers for Disease Control and Prevention’s (CDC’s) Immunization Safety Office and several large US integrated health care systems. The aim of the VSD is to monitor the safety of vaccines routinely administered in the United Sates.^18^ In this observational, case-control, surveillance study conducted from November 1, 2021, to June 12, 2022, we used administrative and electronic health record (EHR) data from 8 sites participating in the VSD to evaluate potential associations between receipt of COVID-19 booster vaccine doses before 20 weeks’ gestation and spontaneous abortion. Primary analyses focused on receipt of a third mRNA COVID-19 vaccine dose in a 28-day exposure window. Secondary analyses evaluated receipt of a booster dose in 28-day or 42-day exposure windows in the full population and limited to the subset who had completed the primary vaccine series. This surveillance was approved by the institutional review boards of all participating sites and the CDC with a waiver of informed consent because this was a minimal risk, observational study and was conducted consistent with federal law and CDC policy. The analytic approach followed the Strengthening the Reporting of Observational Studies in Epidemiology (STROBE) reporting guideline for case-control designs. External researchers can request deidentified data from the VSD for conducting secondary analyses, as described on the VSD website.^19^

### Study Population

Data for this case-control surveillance study came from 8 VSD sites (Kaiser Permanente: Washington, Northwest, Northern California, Southern California, and Colorado; Denver Health; HealthPartners; and Marshfield Clinic). People in the VSD population aged 16 to 49 years with a pregnancy 6 to 19 weeks’ gestation between November 1, 2021, and June 12, 2022, were identified using a validated algorithm applied to automated electronic health data.^20^ The algorithm uses *International Statistical Classification of Diseases, Tenth Revision, Clinical Modification* (*ICD-10-CM*) and *Current Procedural Terminology* (*CPT*) codes from inpatient, outpatient, and emergency department visits, supplemented with clinical data, with updates on a weekly basis, to identify ongoing and completed pregnancies. Ectopic pregnancies, gestational trophoblastic disease, and pregnancies ending in therapeutic abortion were excluded. Pregnancies resulting from assisted reproduction, and thus at increased risk for having a medically attended spontaneous abortion, were also excluded. Exclusions were identified through diagnostic (*ICD-10-CM*) or procedure (*CPT*) codes.

### Surveillance Periods

**Figure 1.**
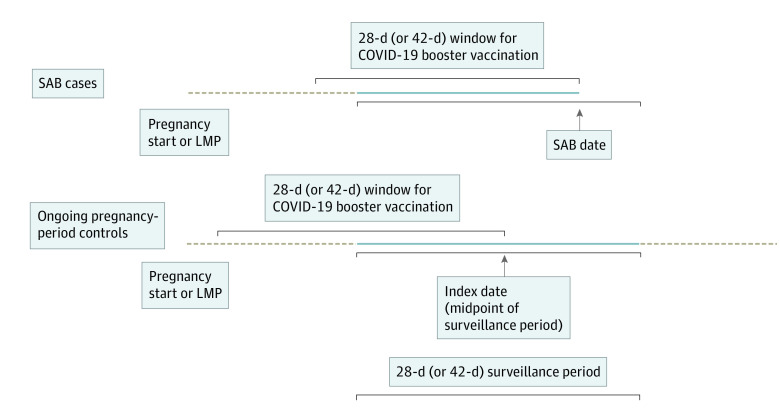
Spontaneous Abortion (SAB) and Ongoing Pregnancy-Period Surveillance and COVID-19 Vaccine Boosters in 28-Day (or 42-Day) Exposure Window Data are stratified by gestational age groups (6-8, 9-13, and 14-19 weeks), maternal age group, number of antenatal visits, race and ethnicity, and Vaccine Safety Datalink site. The dashed beige lines indicate time during pregnancy but outside the surveillance period; the amount of time represented varies by pregnancy and is not to scale with the figure. The solid blue lines represent pregnancy time during the surveillance period. LMP indicates last menstrual period.

Surveillance was conducted from November 2021 through the middle of June 2022. Data presented are from the final data extraction on August 3, 2022. For the primary analyses, evaluating a 28-day vaccine exposure window, eight 28-day surveillance periods were included. For secondary analyses, evaluating a 42-day exposure window, five 42-day surveillance periods were included. Midpoints of the surveillance periods were assigned as the index date for ongoing pregnancies. This index date was then used to assign a gestational age for an ongoing pregnancy period and to evaluate for receipt of a COVID-19 booster vaccination in the prior 28 or 42 days (Figure 1).^21^

### Spontaneous Abortion Cases and Ongoing Pregnancy Controls

Spontaneous abortion cases and ongoing pregnancy controls were identified using a validated algorithm, applied to automated electronic health data. In a previous validation, of 105 spontaneous abortions identified by the algorithm, agreement on pregnancy outcome was 95% and agreement on date of pregnancy outcome was 94%.^20^ Consistent with prior work,^15^ gestational age for both spontaneous abortions and ongoing pregnancies was based on the algorithm’s hierarchical approach to clinical data (last menstrual period and estimated delivery date), gestational age–specific *ICD-10-CM* codes (eg, Z3A.11 for 11 weeks’ gestation), or trimester-specific *ICD-10-CM* codes (eg, O13.2 for gestational hypertension without significant proteinuria, second trimester). Spontaneous abortion cases without gestational age available from these sources were assigned to the earliest gestational age category (6-8 weeks). Ongoing pregnancies with unknown gestational age were excluded.

Spontaneous abortions occurring between 6 and 19 weeks’ gestation were included as cases and assigned to a single surveillance period based on the pregnancy outcome date.^15^ In the primary analyses, during each 28-day surveillance period, eligible ongoing pregnancies between 6 and 19 weeks’ gestation were included as ongoing pregnancy-period controls and assigned an index date equal to the midpoint (day 14) of the 28-day surveillance period. For secondary analyses, using a 42-day surveillance period, the index date was assigned at the midpoint (day 21) of the surveillance period. In both primary and secondary analyses, ongoing pregnancies could be included in more than 1 surveillance period, contributing data as 2 or more ongoing pregnancy-period controls; pregnancies ending in a spontaneous abortion case in 1 surveillance period could contribute data as an ongoing pregnancy-period control in 1 or more surveillance periods before the spontaneous abortion.

### Exposure: COVID-19 Booster Vaccination

As previously described, most vaccines administered in the VSD pregnant population have been the mRNA COVID-19 vaccines, mRNA-1273 (Moderna) or BNT162b2 (Pfizer-BioNTech).^14,22^ A previous case-control surveillance study^15^ of spontaneous abortion after receipt of the COVID-19 primary vaccine series in pregnancy used a 28-day exposure window, consistent with the presumed timing of the inflammatory response after COVID-19 vaccination. As such, primary analyses for the evaluation of booster vaccination in early pregnancy evaluated a third mRNA vaccine (mRNA-1273 or BNT162b2) dose occurring in a 28-day exposure window before the date for the spontaneous abortion (or index date in ongoing pregnancy controls).

In secondary analyses, we evaluated any COVID-19 vaccine booster (including a second dose of Ad26.COV.2.S [Janssen] or a second or third dose of an mRNA COVID-19 vaccine after Ad26.COV.2.S or a fourth or fifth mRNA COVID-19 vaccine dose) in a 28-day exposure window. In addition, we evaluated a third mRNA vaccine dose or any COVID-19 vaccine booster in a 42-day exposure window before spontaneous abortion (or index date in ongoing pregnancy controls). Secondary analyses also evaluated associations between COVID-19 booster vaccination and spontaneous abortion among those who had completed the primary vaccine series and thus were booster eligible.

COVID-19 vaccines administered from the start of the COVID-19 vaccine program (December 15, 2020) through June 12, 2022, in the eligible study population were identified from standardized VSD files. The VSD vaccine files include EHR data as well as medical and pharmacy claims and are supplemented through bidirectional communication with regional or state immunization information systems with standardized data quality checks and deduplication of vaccines from multiple sources.^23^ Vaccines were then classified as first, second, third, or subsequent doses. To reduce the potential for vaccine data from different sources (eg, EHR, state immunization registry, and claims) to be counted as distinct doses, we required at least 14 days between dose 1 and dose 2 and at least 28 days between dose 2 and dose 3 or subsequent doses. The median (IQR) time between the second and third mRNA COVID-19 vaccine dose was 252 (220-289) days.

### Covariates

Covariates associated with likelihood of vaccination and risks for spontaneous abortion outcomes were included in models. Maternal age was categorized into the following groups: 16 to 24, 25 to 34, 35 to 39, and 40 to 49 years. Race and ethnicity were based on self-report as documented in the EHR and categorized as Asian, non-Hispanic; Black, non-Hispanic; Hispanic; White, non-Hispanic; or other or unknown (including Hawaiian or other Pacific Islander, Native American or Aleutian, and multiple races). Antenatal health care visits before the spontaneous abortion (or index date for ongoing pregnancy controls) came from EHR data and were classified as 1 or fewer or 2 or more. Gestational week of the spontaneous abortion or index date for ongoing pregnancy-period controls was categorized as 6 to 8, 9 to 13, or 14 to 19 weeks. The VSD site and surveillance period were also included as covariates.

### Statistical Analysis

In primary analyses, we calculated the odds of receiving a third mRNA COVID-19 vaccine in the 28 days before spontaneous abortion for cases compared with the odds of receiving a third mRNA COVID-19 vaccine in the 28 days before an index date for ongoing pregnancy-period controls. Generalized estimating equations with binomial distribution and logit link with robust variance estimates were used to account for unique pregnancies that contributed data in 2 or more surveillance periods and included covariates listed as main factors. Subgroup analyses by manufacturer (Moderna for mRNA-1273 and BioNTech-Pfizer for BNT162b2) were also conducted.

Using this same approach, we calculated adjusted odds ratios (AORs) and 95% CIs for receipt of any COVID-19 booster vaccine in the 28 days before spontaneous abortion or index date in ongoing pregnancy periods. In addition, we evaluated receipt of a third mRNA COVID-19 vaccine or any COVID-19 vaccine booster in the 42 days before spontaneous abortion or index date in ongoing pregnancy periods in secondary analyses. This same approach was applied in secondary analyses for the subset who were booster eligible, having completed the primary COVID-19 vaccine series. All analyses were performed using SAS/STAT software, version 9.4 (SAS Institute Inc).

## Results

A total of 112 718 unique pregnancies (mean [SD] maternal age, 30.6 [5.5] years; 100% women; 15.1% Asian, non-Hispanic; 7.5% Black, non-Hispanic; 35.6% Hispanic; 31.2% White, non-Hispanic; and 10.6% other or unknown race or ethnicity) were included in the study, with 14 226 pregnancies (12.6%) ending in a spontaneous abortion. The primary analyses included a total of 285 079 pregnancy periods (14 226 spontaneous abortion cases and 270 853 ongoing pregnancy-period controls), with 11 648 (4.1%) having received a third mRNA vaccine in a 28-day exposure window. Across both cases and controls, receipt of a third mRNA vaccine dose in a 28-day window varied by race and ethnicity (2775 [6.5%] in Asian, non-Hispanic people, 495 [2.3%] in Black, non-Hispanic people, 3276 [3.2%] in Hispanic people, and 4323 [4.9%] in White, non-Hispanic pregnant people) (Table 1).

**Table 1.** Pregnant Populations Included in 28-Day and 42-Day Surveillance Periods and Receipt of Third COVID-19 mRNA Vaccine Dose by Selected Characteristics, November 1, 2021, to June 12, 2022^a^

Characteristic	Eight 28-d surveillance periods		Five 42-d surveillance periods	
	No.	Third COVID-19 mRNA vaccine dose, No. (%)	No.	Third COVID-19 mRNA vaccine dose, No. (%)
All	285 079	11 648 (4.1)	182 025	11 669 (6.4)
Spontaneous abortion	14 226	553 (3.9)	13 326	861 (6.5)
Ongoing pregnancy periods^b^	270 853	11 095 (4.1)	168 699	10 808 (6.4)
Maternal age group, y				
16-24	40 600	654 (1.6)	25 875	618 (2.4)
25-34	176 177	7395 (4.2)	112 163	7339 (6.5)
35-39	55 865	3000 (5.4)	35 867	3058 (8.5)
40-49	12 437	599 (4.8)	8120	654 (8.1)
Race and ethnicity				
Asian, non-Hispanic	42 837	2775 (6.5)	27 288	2802 (10.3)
Black, non-Hispanic	21 177	495 (2.3)	13 585	490 (3.6)
Hispanic	102 202	3276 (3.2)	65 148	3249 (5.0)
White, non-Hispanic	88 550	4323 (4.9)	56 656	4021 (7.1)
Unknown or other^c^	30 313	1099 (3.6)	19 348	1107 (5.7)
Gestational age group, wk				
6-8	66 576	2252 (3.4)	43 556	2644 (6.1)
9-13	103 211	3802 (3.7)	65 894	3652 (5.5)
14-19	115 292	5594 (4.9)	72 575	5373 (7.4)
No. of antenatal visits				
≤1	105 726	3464 (3.3)	67 475	3624 (5.4)
≥2	179 353	8184 (4.6)	114 550	8045 (7.0)

^a^ The 28-day surveillance periods were November 1 to 28, 2021; November 29 to December 26, 2021; December 27 to January 23, 2022; January 24 to February 20, 2022, February 21 to March 20, 2022; March 21 to April 27, 2022; April 28 to May 15, 2022; and May 16 to June 12, 2022. The 42-day surveillance periods were November 1 to December 12, 2022; December 13, 2021, to January 23, 2022; January 24 to March 6, 2022; March 7 to April 17, 2022; and April 18 to May 29, 2022.

^b^ Unique ongoing pregnancies may be counted in more than 1 surveillance period and were identified at the midpoint of each 28-day or 42-day period (day 14 or day 21, respectively).

^c^ Other includes American Indian or Aleutian, Hawaiian or other Pacific Islander, and multiple races.

Of 270 853 ongoing pregnancy-period controls, 11 095 (4.1%) received a third mRNA COVID-19 vaccine dose within 28 days of their index date, whereas 553 of 14 226 cases (3.9%) ending in spontaneous abortion received a third mRNA COVID-19 vaccine dose within 28 days of the spontaneous abortion. Although third mRNA vaccine doses were noted in every surveillance period, most were during the first four 28-day surveillance periods (Table 2).

**Table 2.** Receipt of Third mRNA COVID-19 Vaccine Dose in 28-Day Exposure Window Among Ongoing Pregnancy Controls and Spontaneous Abortion Cases by 28-Day Surveillance Period, November 1, 2021, to June 12, 2022

Surveillance period	Dates	Ongoing pregnancies^a^		Spontaneous abortions^b^	
		No.	Third mRNA COVID-19 vaccine dose, No. (%)	No.	Third mRNA COVID-19 vaccine dose, No. (%)
1	Nov 1, 2021-Nov 28, 2021	34 726	1674 (4.8)	1665	74 (4.4)
2	Nov 29, 2021-Dec 26, 2021	34 317	2509 (7.3)	1730	133 (7.7)
3	Dec 27, 2021-Jan 23, 2022	34 339	2736 (8.0)	1769	136 (7.7)
4	Jan 24, 2022-Feb 20, 2022	34 331	2305 (6.7)	1877	119 (6.3)
5	Feb 21, 2022-Mar 20, 2022	34 255	974 (2.8)	1802	49 (2.7)
6	Mar 21, 2022-Apr 17, 2022	34 018	455 (1.3)	1778	25 (1.4)
7	Apr 18, 2022-May 15, 2022	33 087	256 (0.8)	1812	5 (0.3)
8	May 16, 2022-Jun 12, 2022	31 780	186 (0.6)	1793	12 (0.7)

^a^ Unique pregnancies may contribute data for more than 1 surveillance period; eligible ongoing pregnancies of 6 to 19 weeks’ gestation are included in each surveillance period.

^b^ Spontaneous abortion cases occurring from 6 to 19 weeks’ gestation were assigned to a single surveillance period based on the outcome date. Pregnancies ending in a spontaneous abortion case in 1 surveillance period could contribute data as an ongoing pregnancy-period control in 1 or more prior surveillance periods.

**Figure 2.**
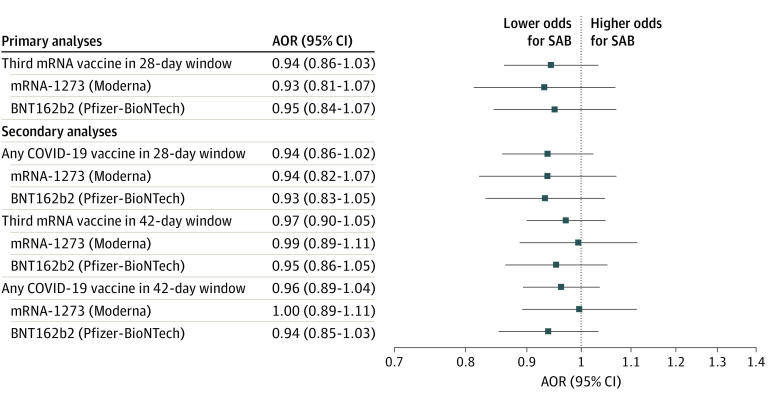
Adjusted Odds Ratios (AORs) for Primary and Secondary Analyses, COVID-19 Booster Vaccination, and Spontaneous Abortion (SAB) at 8 Vaccine Safety Datalink Sites, November 1, 2021, to June 12, 2022 Generalized estimating equation models included gestational age group, surveillance period, maternal age group, number of antenatal visits, site, and race and ethnicity factors and accounted for unique pregnancies that included multiple pregnancy periods. mRNA indicates messenger RNA.

In primary analyses, receipt of a third mRNA COVID-19 vaccine was not associated with spontaneous abortion (AOR, 0.94; 95% CI, 0.86-1.03) (Table 3). Results were consistent when stratified by vaccine manufacturer (mRNA-1273: AOR, 0.93; 95% CI, 0.81-1.07; and BNT162b2: AOR, 0.95; 95% CI, 0.84-1.07) (Figure 2).

**Table 3.** Adjusted Odds Ratios for Receipt of Third COVID-19 mRNA Vaccine in a 28-Day Exposure Window Across 8 Vaccine Safety Datalink Sites, November 1, 2021, to June 12, 2022

COVID-19 mRNA vaccine exposure	Ongoing pregnancy-period controls		Spontaneous abortion cases		Adjusted odds ratio (95% CI)^a^
	No.	COVID-19 vaccine booster, No. (%)	No.	COVID-19 vaccine booster, No. (%)	
**Primary analysis**					
Third mRNA COVID-19 vaccine in 28-d window	270 853	11 095 (4.1)	14 226	553 (3.9)	0.94 (0.86-1.03)
**Secondary analyses**					
Any COVID-19 booster vaccine in 28-d window	270 853	11 952 (4.4)	14 226	592 (4.2)	0.94 (0.86-1.02)
Third mRNA COVID-19 vaccine in 42-d window	168 699	10 808 (6.4)	13 326	861 (6.5)	0.97 (0.90-1.05)
Any COVID-19 vaccine booster in 42-d window	168 699	11 579 (6.9)	13 326	915 (6.9)	0.96 (0.89-1.04)

^a^ Generalized estimating equation models included gestational age group, surveillance period, maternal age group, number of antenatal visits, site, and race and ethnicity factors and accounted for unique pregnancies that included multiple pregnancy-periods.

### Secondary Analyses

#### Any COVID-19 Booster in a 28-Day Window

Among 270 853 ongoing pregnancy-period controls, 11 952 (4.4%) received any COVID-19 booster dose within 28 days of their index date, whereas 592 (4.2%) of 14 226 cases received any COVID-19 vaccine booster dose in a 28-day window before the spontaneous abortion. In these analyses, among 12 544 booster doses administered to cases and controls, 11 701 (93.3%) were third doses, 698 (5.6%) were second doses, 135 (1.1%) were fourth doses, and 10 (0.1%) were fifth doses. Receipt of any COVID-19 booster vaccination within a 28-day window was not associated with spontaneous abortion (AOR, 0.94; 95% CI, 0.86-1.02) (Table 3), with results consistent when stratified by vaccine manufacturer (mRNA-1273: AOR, 0.94; 95% CI, 0.82-1.07; and BNT1262b2: AOR, 0.93; 95% CI, 0.83-1.05) (Figure 2).

#### Additional Secondary Analyses

Secondary analyses included 103 156 unique pregnancies across five 42-day surveillance periods from November 1, 2021, to May 29, 2022; 89 830 (87.1%) remained ongoing pregnancies and 13 326 (12.9%) ended in a spontaneous abortion. Secondary analyses using a 42-day surveillance period included a total of 182 025 pregnancy periods (13 326 cases and 168 699 ongoing pregnancy-period controls), with 11 669 (6.4%) having received a third mRNA vaccine and 12 494 (6.9%) having received any COVID-19 vaccine booster in a 42-day exposure window. Receipt of a third mRNA COVID-19 vaccine (AOR, 0.97; 95% CI, 0.90-1.05) or any COVID-19 vaccine booster (AOR, 0.96; 95% CI, 0.89-1.04) was not associated with spontaneous abortion (Table 3). Results were consistent when stratified by vaccine manufacturer (Figure 2). In secondary analyses limited to those who were booster eligible, there was no association between COVID-19 booster vaccination and spontaneous abortion, consistent with results for the full cohort (eTable in Supplement 1).

## Discussion

In this large case-control surveillance study of more than 100 000 unique pregnancies and analyses that included 285 079 pregnancy periods, receipt of a COVID-19 booster vaccine in early pregnancy was not associated with spontaneous abortion. Our primary analyses focused on the receipt of a third mRNA COVID-19 vaccine in a 28-day exposure window because this was the most common type of booster vaccination in our population. Our findings were consistent in secondary analyses that evaluated receipt of any COVID-19 booster, the 42-day exposure window, and associations in the subset who had completed the primary vaccine series.

The need to monitor the safety of booster vaccination in early pregnancy is clear, given the potential for repeat vaccine doses to be associated with local and systemic symptoms.^24^ Furthermore, concerns regarding reactogenicity of booster vaccination may be contributing to vaccine hesitancy.^24^ However, data to date have not demonstrated that COVID-19 booster vaccination is associated with an increase in acute reactions in pregnant or other adult populations. A prospective study^24^ of more than 17 000 adults receiving a COVID-19 booster vaccine, including 2009 who were pregnant at the time of vaccination, found that the rates of self-reported local and systemic reactions after COVID-19 booster vaccination were similar to those following a second COVID-19 vaccine dose. As of March 24, 2022, the Vaccine Adverse Event Reporting System had received 323 reports of adverse events in pregnant people after a COVID-19 booster vaccine. However, the overall safety profile was similar to that after the initial COVID-19 vaccine series.^25^ In addition, v-Safe, a voluntary after-vaccination health checker, found that individuals 18 years or older reported fewer injection site or systemic reactions after a first booster dose than after dose 1 or dose 2 of the primary series.^26^ Similarly, our surveillance study of third mRNA vaccine doses and spontaneous abortion found an AOR of 0.94 (95% CI, 0.86-1.03), consistent with our findings following the first or second mRNA vaccine dose (AOR, 1.02; 95% CI, 0.96-1.08).

### Strengths and Limitations

This study has several strengths, including the use of a large and diverse population-based sample, the availability of comprehensive data on COVID-19 vaccine exposures,^23^ and the ability to monitor associations between vaccine exposures in early pregnancy and spontaneous abortion with data and analyses updated on a monthly basis.^27^ However, several limitations to these analyses should be noted. First, spontaneous abortion cases were identified and assigned a gestational age based on automated electronic health data by using a validated algorithm. The algorithm shows substantial agreement with manual record review for distinguishing pregnancies ending in live birth from pregnancies ending in spontaneous abortion. However, for pregnancies ending in spontaneous abortion, the agreement between the algorithm assigned and record review gestational age might be lower.^20^ Errors in algorithm-derived gestational age assignment could result in misclassification of vaccine exposure status, biasing results to the null, especially when applying a 28-day exposure window. It was reassuring that results were consistent in secondary analyses using a 42-day exposure window.

Second, all included spontaneous abortion cases were medically attended and estimated to have reached at least 6 weeks’ gestation. These requirements are consistent with definitions applied in prior maternal vaccine safety surveillance studies^15,28,29^ and with American College of Obstetricians and Gynecologists case definitions.^30^ Furthermore, multiple-gestation pregnancies with a single fetal demise could not be excluded. Alternate approaches would be needed (eg, prospective design and active participant enrollment) to analyze all cases of early pregnancy loss.

Third, although we included available covariates, including maternal age group and number of antenatal visits, as main factors in the analyses, we did not have data on other important confounders, such as maternal educational level, prior history of spontaneous abortion, maternal body mass index, or recent COVID-19 infection. Residual confounding is a potential limitation of observational studies of maternal vaccination.^27^

Fourth, our primary analyses focused on a third mRNA vaccine dose (after 2 prior mRNA vaccines) because this was the most common booster vaccine type used in VSD during the surveillance period. We did not have sufficient mRNA COVID-19 vaccine doses administered in pregnant people after prior receipt of the Janssen vaccine or second doses of the Janssen vaccine to specifically evaluate these exposures, but they were included in the secondary analyses of any COVID-19 vaccine booster. Furthermore, these analyses predated availability of the bivalent COVID-19 booster vaccine.

### Conclusions

In summary, compared with ongoing pregnancies, the odds of having received a COVID-19 booster vaccination in a 28- or 42-day exposure window before spontaneous abortion were not increased. These findings support the safety of recommendations for COVID-19 booster vaccination, including in pregnant populations. As of September 1, 2022, bivalent COVID-19 booster vaccines have been approved and recommended for use in the United States.^31^ Studies of bivalent booster vaccine exposures in early pregnancy are ongoing and will be important.

## Notes

Supplement 1. **eTable.** Secondary Analyses, Adjusted Odds Ratios and 95% Confidence Intervals Among Pregnant People Eligible for a COVID-19 Booster Dose, by 28-Day and 42-Day Exposure Windows and by Manufacturer

Supplement 2.
Data Sharing Statement
