# Supplementary material for: COVID-19 Booster Vaccination in Early Pregnancy and Surveillance for Spontaneous Abortion
Source: JAMA Netw Open. 2023 May 19;6(5):e2314350. doi: 10.1001/jamanetworkopen.2023.14350 (PMC10199343; doi:10.1001/jamanetworkopen.2023.14350)
Supplement: Supplement 1. — eTable. Secondary Analyses, Adjusted Odds Ratios and 95% Confidence Intervals Among Pregnant People Eligible for a COVID-19 Booster Dose, by 28-Day and 42-Day Exposure Windows and by Manufacturer [file jamanetwopen-e2314350-s001.pdf]

Supplementary Online Content

Kharbanda EO, Haapala J, Lipkind HS, et al. COVID-19 booster vaccination in early pregnancy and surveillance for spontaneous abortion. *JAMA Netw Open*. 2023;6(5):e2314350. doi:10.1001/jamanetworkopen.2023.14350

eTable. Secondary Analyses, Adjusted Odds Ratios and 95% Confidence Intervals Among Pregnant People Eligible for a COVID-19 Booster Dose, by 28-Day and 42-Day Exposure Windows and by Manufacturer

This supplementary material has been provided by the authors to give readers additional information about their work.

© 2023 Kharbanda EO et al. *JAMA Network Open.*

eTable. Secondary Analyses, Adjusted Odds Ratios and 95% Confidence Intervals Among Pregnant People Eligible for a COVID-19 Booster Dose,[a] by 28-Day and 42-Day Exposure Windows and by Manufacturer

**Vaccine type and exposure window**	**Unique pregnant people in analyses**	**Adjusted Odds Ratios[b] (95% CI)**
3rd mRNA vaccine in 28-day window	68,900	0.97 (0.88 - 1.06)
mRNA-1273, Moderna	68,317	0.95 (0.83 - 1.09)
BNT162b2, Pfizer-BioNTech	68,532	0.98 (0.87 - 1.10)
Any COVID-19 vaccine in 28-day window	73,153	0.96 (0.88 - 1.06)
mRNA-1273, Moderna	72,529	0.96 (0.84 - 1.10)
BNT162b2, Pfizer-BioNTech	72,749	0.96 (0.85 - 1.08)
3rd mRNA vaccine in 42-day window	49,419	0.98 (0.90 - 1.06)
mRNA-1273, Moderna	48,052	0.96 (0.85 - 1.08)
BNT162b2, Pfizer-BioNTech	48,538	1.00 (0.90 - 1.11)
Any COVID-19 vaccine in 42-day window	52,425	0.96 (0.89 - 1.04)
mRNA-1273, Moderna	50,970	0.96 (0.85 - 1.07)
BNT162b2, Pfizer-BioNTech	51,464	0.99 (0.89 - 1.09)

[a]Eligible for a COVID-19 Booster dose defined as: 1) For analyses of 3rd mRNA vaccine, 2 initial mRNA doses received, with 2nd dose between 14 and 56 days from first. Eligible 3rd mRNA doses were required to be at least 60 days after the 2nd mRNA dose. For unvaccinated, the exposure window was required to be at least 60 days after 2nd dose. 2) For analyses of any COVID-19 vaccine booster, additional booster eligible population included those whose initial COVID-19 vaccine dose is J&J/Janssen. For vaccinated, their booster is a 2nd J&J/Janssen dose or 1st mRNA COVID-19 dose at least 60 days after dose 1. For unvaccinated, exposure window is at least 60 days after 1st dose.
[b] Generalized estimating equation (GEE) models included gestational age group, surveillance period, maternal age group, number of antenatal visits, site, and race and ethnicity factors and accounted for unique pregnancies that included multiple pregnancy-period.

© 2023 Kharbanda EO et al. *JAMA Network Open.*
