# Supplementary material for: COVID-19 Booster Vaccination in Early Pregnancy and Surveillance for Spontaneous Abortion
Source: JAMA Netw Open. 2023 May 19;6(5):e2314350. doi: 10.1001/jamanetworkopen.2023.14350 (PMC10199343; doi:10.1001/jamanetworkopen.2023.14350)
Supplement: Supplement 2. — Data Sharing Statement [file jamanetwopen-e2314350-s002.pdf]

Data Sharing Statement

Kharbanda. COVID-19 Booster Vaccination in Early Pregnancy and Surveillance for Spontaneous Abortion. *JAMA Netw Open*. Published May 19, 2023. doi:10.1001/jamanetworkopen.2023.14350

Data
Data available: Yes
Data types: Other (please specify)
Additional Information: Data from the Vaccine Safety Datalink can be requested at the URL listed below
How to access data: https://www.cdc.gov/vaccinesafety/ensuringsafety/monitoring/vsd/accessing-data.html
When available: With publication

Supporting Documents
Document types: Statistical/analytic code
How to access documents: https://www.cdc.gov/vaccinesafety/ensuringsafety/monitoring/vsd/accessing-data.html
When available: With publication

Additional Information
Who can access the data: The VSD data sharing program is administered by the National Center for Health Statistics Research Data Center (NCHS RDC). VSD data created before 2000 are available through the data sharing program for new vaccine safety studies for analyses at the RDC Datasets from published VSD studies from 2002 to the present may be accessed for secondary analyses at the RDC The VSD data sharing program is a three-step process: Submisson of proposals to NCHS RDC Submission of proposal to VSD participating sites' Institutional Review Boards (IRBs) Access to VSD data The VSD data available through this data sharing program can only be accessed at the NCHS RDC. Specific details for this program can be found at 'Guidelines for VSD Data Sharing'
Types of analyses: For evaluation of vaccine safety
Mechanisms of data availability: After approval of a proposal
Any additional restrictions: As above
